# Occupational therapy students' perceptions of their experience in a role-emerging Level II fieldwork within higher education student services

**DOI:** 10.1186/s12909-024-05303-7

**Published:** 2024-04-08

**Authors:** Marie-Christine Potvin, Alexis N. Morales, Erin K. West, Mika Kalimi, Jeanne M. Coviello

**Affiliations:** 1 Department of Occupational Therapy, Thomas Jefferson University, 901 Walnut Street, 6th Floor, Philadelphia, PA 19107 USA; 2 College of Public Health, Temple University, Mitten Hall, Room 201E, 1913 North Broad Street, Philadelphia, PA 19122 USA

**Keywords:** Fieldwork, Clinical rotation, Experimental Learning, Occupational Therapy, Role-Emerging, Non-Traditional

## Abstract

**Background:**

Role-emerging settings – those where occupational therapy (OT) services have not traditionally been provided – are common sites for practice placements of entry-level occupational therapy students. A growing body of literature has attempted to determine the value and drawbacks of such practice placements on the professional preparedness of OT students with mixed findings. Benefits have been identified, including increased cultural understanding, advocacy, creativity, initiative, and problem-solving skills. However, OT students have been reported to perceive such placement as limiting their professional growth and preparedness to practice compared to traditional placements.

**Methods:**

A phenomenological study was conducted seeking the perceptions of OT students (*n* = 14) about their clinical placement at a role-emerging site. Recorded semi-structured interviews were conducted by trained interviewers within two weeks of the end of clinical placement. The recordings were transcribed verbatim and then coded using an iterative multi-coder inductive approach. Inter-coder agreement, reflectivity, and audit trail were maintained.

**Results:**

Three themes emerged from the analysis: (1) integrating independence and support, (2) becoming occupational therapists, and (3) filling a gap. These themes reflect students’ positive perceptions of their role-emerging clinical placement. They felt that this placement allowed them to develop self-confidence and professional identity as occupational therapists and learn new skills while simultaneously filling a gap in services for clients. Most importantly, they felt that this placement prepared them for their future OT practice.

**Conclusion:**

This finding and their resounding support of the experience suggest that OT students can perceive role-emerging placement as a solid foundation for clinical practice. Factors, included in this placement, that may have contributed to their experience include the level of support provided, time available for learning including space to make mistakes, and freedom from productivity and payor requirements.

## Background

Practice placements are mandatory for occupational therapy education programs to be recognized as meeting the minimum standard by the World Federation of Occupational Therapy [1]. In the United States (US), practice placements for master and doctoral degree occupational therapy students are known as fieldwork and are divided into Level I and Level II, with the latter being a more substantial experience (i.e., typically full-time for 12 weeks; Accreditation Council for Occupational Therapy Education [2]. Level II fieldwork provides students with opportunities for practical application of newly acquired occupational therapy knowledge and skills under the guidance of a licensed occupational therapist within traditional or non-traditional settings [2]. Occupational therapy practice settings categorized as “traditional” are those where the role of occupational therapy is already clearly established. In the US, these include hospitals, outpatient clinics, schools, and skilled nursing facilities. Any occupational therapy practice outside of these areas is labeled “non-traditional” or “role-emerging” to indicate settings where occupational therapy services are being developed [1, 3, 4].

In recent years, in the United States, there has been a rise in occupational therapists branching out of traditional occupational therapy practice and establishing occupational therapy services in role-emerging settings such as county jails, drug and alcohol rehabilitation centers, and universities [4–9]. This shift from traditional to role-emerging practice has been attributed to both economic and philosophical principles that allow clinicians to provide holistic care to address clients’ health, wellness, and quality of life while not being dependent on third-party reimbursement [10]. As a result, there is an increased demand for occupational therapists who are skilled to work in these settings [6–9]. In parallel, there is thus a growing need to train future occupational therapists to be competent in role-emerging practice, which can be accomplished, in part, by providing fieldwork experiences in such settings [6, 7, 11–14]. In fact, some have stated that providing training to occupational therapy students in role-emerging practice settings is imperative to the profession [6, 13, 15]. This mirrors what has been documented recently in the US with an increase in the use of role-emerging settings for fieldwork in response to the increase in the need for sites as more occupational therapy programs have been accredited [8, 16].

Both traditional and role-emerging fieldwork align with US accreditation standards and provide FW students with exposure to persons and/or groups across the lifespan with varying conditions in settings that reflect current professional practice [2, 3]. Based on review of a wide range of studies, some benefits and challenges of role-emerging fieldwork have been identified. In these settings, students build confidence in the use of occupational therapy skills, increase their cultural understanding, and develop advocacy skills [6, 9, 17]. Furthermore, self-directed learning, which is a hallmark of such placements, results in students experiencing an increase in creativity, initiative, and problem-solving skills [8]. Critical, role-emerging placements were found to promote the development of occupational therapy students’ professional identity [8, 16]. On the other hand, occupational therapy students commonly perceive role-emerging fieldwork placements as lacking the same opportunities for professional growth as traditional placements [6, 8, 9], and in some studies, students reported feeling that role-emerging fieldwork placement failed to prepare them adequately to be competent entry-level practitioners [8, 9]. Studies found that students identified challenges with defining professional roles, anticipated difficulties relating to shared versus individualized supervision and caseloads, experienced strained peer dynamics, limitations within the physical environment, lack of support throughout their placement, taxing physical demands of placement, need for clearly defined expectations and effectiveness of communication between all constituents, and difficulty managing the high level of responsibility [16, 18, 19]. There are thus both benefits and challenges of role-emerging fieldwork placements identified in the literature, however further research on occupational therapy student perceptions of specific types of role-emerging placements is needed to minimize perceived challenges and create beneficial learning environments that facilitate the development of self-confidence and perceived readiness for practice [7–9, 16].

A few higher education institutions in the US have started to use occupational therapy expertise to support the academic success of students with disabilities on their campuses [20–23]. The GOALS^2^ Program intends to enhance the academic attainment of college students with disabilities and therefore, is a novel and under-researched, role-emerging Level II fieldwork placement (Boney et al., 2019, Harrington et al., 2021). The development of this role-emerging fieldwork program was informed by evidentiary literature relating to best practice to maximize the learning experience of the occupational therapy students. For example, a collaborative fieldwork model was used, as past research found that students benefit of peer support and positive peer pressure and value being in pairs or groups to share the lived experience when placed in a role-emerging setting for fieldwork [16, 18]. We intended to explore whether the integration of these practices alleviated occupational therapy students' concerns with being placed in role-emerging settings for fieldwork.

A study was conducted to explore the GOALS^2^ Program Level II occupational therapy fieldwork students’ perceptions of the value of this role-emerging placement on their preparation for clinical practice and their vision of their future practice.

## Methods

An inductive phenomenological qualitative study was conducted to investigate occupational therapy Level II fieldwork student impressions of placement in this role-emerging setting. Phenomenological studies are used to explore the perspective about events or experiences from an individual or a group’s perspective [24], which matches the intent of this study. An inductive method was used, as the analysis was conducted without the use of a preexisting coding frame, frame of reference, or the researchers' preconceptions [25].

### Participants

The cohort of occupational therapy students (*n* = 14), who completed their Level II fieldwork with the GOALS^2^ Program, were invited to participate in the study. We projected that 9–12 interviews would allow a point of saturation to be reached. All 14 students who were eligible for the study (hereafter referred to as “participants”) agreed to participate; their choice to participate had no impact on their ability to complete their Level II fieldwork with the program. Participants could withdraw from the study at any time for any reason without consequences. Ethical approval was secured from the university institutional review board prior to the beginning of the study, and all participants signed written consent forms after participating in the informed consent process.

### Fieldwork setting

The GOALS^2^ Program offers free services to university students with disabilities whose needs fall within the occupational therapy scope of practice and were not fully met by pre-existing campus services at a mid-Atlantic private not-for-profit university. The primary approach used within the program is coaching, but services also include assistive technology needs identification and training, adaptation of learning tasks and environments, and advocacy to and education of the clients’ educational units and other students’ services. In addition, the fieldwork students performed administrative tasks related to the range of services provided since they were the primary staff of the program. Finally, the fieldwork students engaged in program development (e.g., attracting clients, bringing awareness of the program within the university, as well as developing and refining intake, documentation, and discharge planning processes), as this was a new program at the inception of the study. All fieldwork students completed at least one project that ranged in type and style (e.g., disability awareness month campaign on campus, spearheading a disability advocacy group on campus, presentation about person-first language and disability-supportive interactions for safety and security personnel). The program’s location on campus was a small conference room on the main campus, although services were provided in different locations based on need. Services were mostly offered Monday through Friday during regular business hours, with flexibility to meet clients’ needs.

Two Level II fieldwork students supported the program each semester with supervision from two occupational therapists onsite for a total minimum of eight hours per week (range: 8–16 h). In addition, at least one occupational therapist was always available via email, text, or phone for time-sensitive needs. Finally, the Level II occupational therapy fieldwork students had access to the support of the director of the students’ accessibility office and the dean of students if needed. One of the supervising occupational therapists had four years of clinical experience primarily in community mental health and the second 20 years of experience primarily in pediatrics at the beginning of the study. Both had limited experience as fieldwork educators.

The occupational therapy fieldwork students with the GoALS^2^ Program who became participants in this study completed the didactic portion of their occupational therapy education in one of three programs (i.e., two master-level and one doctorate-level) within one university. This was the same university where the clients of the GOALS^2^ Program were students. The participants had completed their Level I fieldwork experiences at a variety of sites, and all had a role-emerging Level I fieldwork experience. The participants were either completing their first or second Level II fieldwork experience with the GOALS^2^ Program. Data for this study were collected within 2 weeks of the end of their Level II fieldwork with the Program. As commonly used in role-emerging fieldwork, the GOALS^2^ Program uses a collaborative fieldwork model, where a group of students actively learn and work together on all aspects throughout their experience [3, 16].

### Research team

The research team was composed of five authors, and their work was supported by several graduate assistants. All members of the research team self-identified as female and white; one self-identified their ethnicity as Hispanic/Latina. All research team members were occupational therapists (1st and 5th authors) or occupational therapy students (authors 2–4) at the time of the study. The first and fifth authors had experience in qualitative and quantitative research methodologies. The 1st author was an occupational therapy faculty member at the university and the administrator and one of the fieldwork supervisors of the GOALS^2^ Program. To minimize the potential effect of the 1st author’s multiple roles with the participants, safeguards were put in place: (1) the informed consent process was completed by the graduate assistants; (2) the data analysis was completed by the 2nd and 3rd authors with the 1st author’s role confined to discrepancy resolution. Authors 2–3 completed their doctoral experiential and capstone project within the GOALS^2^ Program during the academic years that followed the end of the data collection for this study. The fifth author has extensive experience in fieldwork coordination and role-emerging fieldwork but was not involved with the GOALS^2^ Program beyond this study. The graduate assistants were not involved with the GOALS^2^ Program beyond the study.

### Data collection

**Fig. 1:**
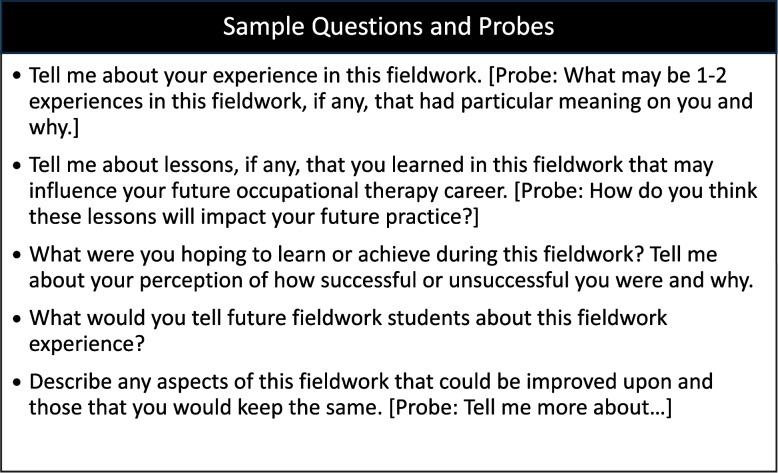
Semi-structured interview guide used in the study

The data for the study were collected through semi-structured interviews conducted in person or via Zoom by one of the trained graduate assistants using an interview guide (see Fig. 1). The interviews ranged in length from 30 to 45 min. To increase the study results’ trustworthiness and minimize potential bias, an audit trail, decision-making processes, and reflexive journals, to document self-evaluation of insights, personal reflection, and provide context for researchers’ reactions, were maintained throughout the data collection and data analysis process [26]. All data, audio recordings, and text files were saved on password-protected servers and accessed through password-protected computers by study personnel only.

### Data analysis and preparation

All interviews were audio-recorded, transcribed verbatim using a transcription protocol, checked for reliability, and de-identified to prepare the data for analysis by trained graduate research assistants who did not conduct the interviews. The data were then analyzed in a multi-step iterative process that included developing the codebook, coding, and deductive interpretation.

### Codebook development

The inductive approach, described by [27], was utilized to develop the codebook using an iterative multi-coder process involving (1) reading the interview transcripts to identify potential codes and code definitions to capture the interviewees’ messages; (2) adjusting codes, definitions, and hierarchies as more transcripts were read; and (3) calibrating the hierarchically organized codes until no new insights or understandings emerged and saturation was achieved. This process was completed by two researchers (the second and third authors) via discussion of rationale until codes were reliably coded using the definitions. They discussed their approach, codes, and interpretation with the lead researcher (the first author) throughout the process.

### Data coding

Once the codebook was finalized, all transcripts were uploaded into NVivo (Version 12). The second and third authors coded all 14 transcripts independently and then compared their coding to establish intercoder reliability across all transcripts, achieving ~ 95% intercoder agreement. Any differences in coding were resolved through discussion with the lead author.

### Data interpretation

Once all interviews were coded, the second and third authors independently identified themes using a non-linear, phenomenological, narrative inquiry process in which the data were organized by codes to illuminate themes and then conducted peer debriefing [26]. They reviewed the audit trails and reflexive journals to ensure that their interpretation had not missed key elements. Expert debriefing with the lead researcher (first author) occurred to confirm the rigor and interpretation of the analysis. Interpretation of data was confirmed via member checking with four past fieldwork students involved with the GOALS^2^ Program.

## Results

The participants’ socio-demographic information, collected verbally and by email, is summarized (see Table 1). These variables were selected to describe the participants to ascertain the transferability of results for this qualitative study. Thirteen of the participants identified as women and as white, which is reflective of the degree of diversity within the US occupational therapy workforce [27]. The mean age of the participants was 30.6 years old (SD = 7.6). Of the 14 participants, 50% reported that occupational therapy was not their first career. This was expected as one of the occupational therapy programs from which the Level II students were drawn was hybrid and intended for non-traditional students.

**Table 1:** Participant’s sociodemographic information (*n* = 14)

Characteristics	Frequency (*n*)	Percentage (%)
Gender		
Woman	13	92.9
Man	1	7.1
Age (years)		
20–29	8	57.2
30–39	4	28.6
40–49	2	14.2
Ethnicity/Race		
White	13	92.9
Black or African American, Hispanic or Latino,	1	7.1
Native American, Asian		
Level II fieldwork in partial fulfillment of:		
Master of Occupational Therapy	12	85.7
Occupational Therapy Doctorate	2	14.3
Highest degree held prior to beginning their occupational therapy program		
Bachelor’s degree	13	92.9
Master’s degree	1	7.1

**Fig. 2:**
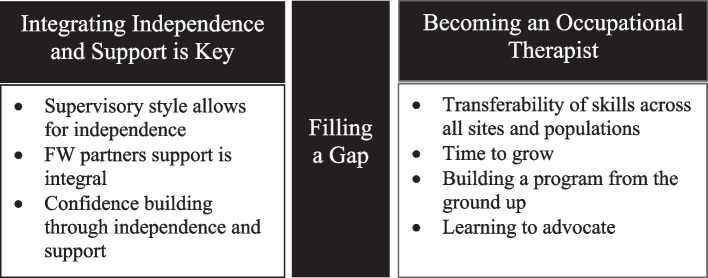
Themes and subcomponents that emerged from the study

The qualitative analysis identified three major themes: *Integrating Independence and Support is Key*, *Becoming an Occupational Therapist*, and *Filling a Gap,* and seven subcomponents (see Fig. 2). In the theme *Integrating Independence and Support is Key* students spoke of the importance of both being autonomous in their evaluation and treatment of clients while simultaneously having a support system through their peer partner and fieldwork educators. *Becoming an Occupational Therapist* captured participants’ perceptions of the spectrum of skills that they developed during this placement, which they believed were both uniquely gained in a role-emerging setting and fundamental to occupational therapy practice. Last, the theme *Filling a Gap* emerged from fieldwork students’ perception of the impact that they made on this university campus by providing necessary services to address any barrier faced by their clients, including but not limited to, academic achievements such as social participation and community mobility. These themes and their subcomponents are further described and illustrated through exemplary quotes in the following paragraphs.

### Integrating independence and support is key

Most participants (13/14) spoke of the need to be self-directed in this fieldwork setting because of the distant supervision model. Participants also spoke of the collaborative learning model used, and its importance in gaining independence while feeling supported. Participants reflected on the importance of the support they received from their fieldwork educators, as well as from their peer partner, which allowed them to build their independence. Participants explained how independence and support came together allowing them to gain greater confidence in themselves, contributing to the development of their professional identity as occupational therapists. The participants emphasized the effectiveness of this dual approach, and within this broad theme, three subcomponents emerged: supervisory style allows for independence, fieldwork partner support is integral, and confidence building through independence and support.

### Supervisory style allows for independence

All but one of the participants credited support from their fieldwork educators, which came in the form of trust, availability, and weekly supervisory meetings, for fieldwork placement success. Participants felt that the fieldwork educators trusted them to get the work done. One participant noted, “there was a lot of independence and… trust in us as students to move forward and to handle things. That was great” (P7, L436-437). Even with distant supervision, participants never felt alone. A participant noted, “Both [fieldwork educators] provided excellent preceptorship. The amount of time that they spent with us was adequate. They were supportive. They gave you responsibility. I wouldn’t change that. It felt like the right balance of independent responsibility and leadership” (P12, L182-189). Participants also felt that their fieldwork educators were available to them outside the designated eight hours of supervisory time. One participant stated, “I always felt [that] if I had a question, I could go to her” (P1, L477-481). The structure of weekly meetings to debrief and discuss caseloads with the fieldwork educators allowed students not to feel isolated. In addition, it provided an opportunity to cultivate professional skills. “We had weekly clinical meetings that were really effective. [We] touched base and saw where we were. It really helped develop our professional reasoning” (P1, L449-451).

### Fieldwork partner support is integral

Of the participants, 12/14 identified the collaborative learning model as another major contributor to their success. They found that having a fieldwork partner at the site was an asset. In the participants’ words, “the multiple fieldwork students’ dynamic is really important” (P4, L221). Participant Four stated, “having less hands-on supervisor time, it's important to have someone you can bounce ideas off of…and just work closely with so you’re not alone” (L221-223). Partners were able to complement each other's strengths and function as a team. A participant stated, "a total benefit for us was that me and my partner had two different skill sets. So, we were able to complement each other very well” (P12, L220-222). When reflecting on their future practice, participants expressed that working so closely with a peer mirrored the team approach in many other settings, and it served them well to develop those skills in this fieldwork experience. Participant Fourteen stated, “the idea of having to work in a team and make those decisions as a partnership was really valuable… because [in future practice] we never are going to be making our decisions alone, even though we might be treating alone” (L507-509).

### Confidence building through independence and support

Participants appreciated that the supervision and collaborative learning model of this placement which they felt promoted their autonomy and increased self-confidence. A participant noted, “[I found] my independence and my confidence on my own, without someone holding my hand” (P2, L435-436). Clients in the GOALS^2^ Program are typically in stable health with no immediate safety risks or medical complications, which creates a lower-pressure environment. Therefore, participants were apt to independently make mistakes and learn from them, a process that helped them gain self-confidence. Participant Four notes, “I think starting out with a slightly more low-pressure setting was really helpful in building my self-confidence” (L179-181).

### Becoming an occupational therapist

All participants spoke about the skills gained through their time in this fieldwork and how it contributed to their development as occupational therapists and would be valuable in their future practice. They valued the opportunities to develop skills outside of those typically learned during fieldwork. For example, during this fieldwork placement, participants learned professional coaching techniques which they employed with clients. Further, their projects and the administration of the GOALS^2^ Program led them to discover a new passion for advocacy and develop an understanding of program development. Four subcomponents emerged as participants shared the role of this fieldwork placement in their development as occupational therapists: transferability of skills across all sites and populations, time to grow, building a program from the ground up, and learning to advocate.

### Transferability of skills across all occupational therapy sites and populations

All of the participants spoke positively about their perception of the transferability of the skills learned in this setting to their next Level II fieldwork and their future careers. Specifically, they mentioned interviewing skills and professionalism as transferable aspects. Referring to the coaching skills learned, one participant stated, “any [occupational therapy] student can benefit from what you learn [with GOALS^2^]… the interactions, interviewing, talking to clients, … they’re all valuable skills… it’s everything you do as an occupational therapist” (P3, L185-192). Participants also mentioned time management as another transferable skill that they developed. Participant Four noted, “not only the communication and professionalism to bring into my future placements but my future career… but also those little things like creating my own schedule and managing my time… as a professional versus a student” (L64-66). When speaking about the development of occupational therapy-specific skills, participants identified building therapeutic relationships as a transferable skill acquired (P12, L117-119) through this specific experience.

### Time to grow

In the host fieldwork placement, occupational therapy students were not held to productivity standards dictated by employers’ or payors’ reimbursement policies. Rather, expectations of workload were incumbent on client needs and fieldwork student readiness. The participants appreciated the amount of time they were afforded to build meaningful therapeutic relationships with their clients. Participant Twelve stated, “the value of the GOALS^2^ Program is that you’re really able to develop therapeutic relationships with people. Other placements don’t just allow you the time to do that” (L157-160). Additional time with clients was attributed to not being bound to productivity markers. One participant stated, “I could develop the skills of doing what was important for the person that I [was] working with versus what an administrator or an insurance company is telling me is important” (P7, L274-282). They added “we were not subject to productivity demand” (P7, L274-282).

### Building a program from the ground up

All participants said that they appreciated being able to hone their program development skills within this fieldwork, an aspect that they were not expecting. Participant Twelve commented, “we performed administrative duties such as scheduling, email correspondence, collaborating and talking with different departments about the program” (L12-14). Regarding the work required to create new systems for the program, one participant remarked, “[we] were working on program development for the program itself, so we actually created a whole discharge process” (P7, L90-91). Participants were tasked not only with running the program but also with continuing to enlarge the program. One participant stated, “one thing that I am proud of is trying to get more clients and expand the program” (P10, L13-14).

### Learning to advocate

All participants repeatedly described having the opportunity to develop skills in advocacy and a “passion to advocate” (P8, L172). Advocacy came in three main forms for participants: collaborating with clients to advocate for themselves, advocating on behalf of their clients, and advocating for the unique value of occupational therapy. In the participants’ view, these advocacy efforts were met with success as well as challenges. Eight of the fourteen participants reflected on how commonly clients wanted support related to self-advocacy. A participant remarked, “I’d say that happened for the majority of our clients” (P8, L176-178). Thus, participants learned how to advocate on behalf of their clients, a skill they mentioned as useful to their future career, as illustrated in this excerpt, “[working with a client to obtain reasonable accommodations] taught me a lot about how we, as occupational therapists, will always need to advocate for our clients” (P5 L129-130). Participants also advocated for the distinct value of occupational therapy while simultaneously building skills in communicating with other members of an interprofessional team. A participant stated, “I grew by collaborating with other offices and having to explain and advocate about the importance of the GOALS^2^ Program [and] occupational therapy” (P10, L181-183). Although advocating was mostly a positive experience, participants had to overcome challenges when university professors demonstrated reluctance to adapt. As one participant commented, “one thing that [was] negative or discouraging is that we have a client that needs accommodations. In her program, there was a lot of push back [against these]. That was pretty eye opening” (P6, L82-91).

### Filling a gap

The last theme, Filling a Gap, does not have subcomponents. Throughout the interviews, participants reflected on the work being done through the GOALS^2^ Program and its potential wider impact on colleges and universities and the scope of occupational therapy practice. While recognizing that accommodations are being offered to college students, participants noted gaps in what is provided that can be filled with services offered by the GOALS^2^ Program. Participant Fourteen reflected, “they had needs that just weren’t being met by all of the supports that were already on campus” (L49-51). The support and services provided by the GOALS^2^ Program give clients more opportunities for success in an environment where they may have otherwise fallen off the radar. A participant remarked, “we’re really filling a gap. There are students whose services dropped off when they graduated high school. When they move to college, they kind of get lost through the cracks” (P1, L135-138).

Filling a gap through occupational therapy service was perceived by participants to be needed not only in terms of supplementing support offered by colleges and universities but also in terms of the major life transition that occurs at that age. Participants recognized the services occupational therapists could provide to support that transition. Participant Eight noted, “it’s such a huge life transition and such an unmet need for all students transitioning to college” (L158-159). This transition could be addressed by occupational therapists and occupational therapy assistants prior to attending university. One participant realized the potential usefulness of this service for high school students, stating, “especially as occupational therapists… how can [we] be a part of that transition? Whether it's socially, academically… the big picture academics, and being successful in that setting, how can we be a part of that?” (P4, L207-209).

## Discussion

Studies have found that occupational therapy fieldwork students learn similar skills in role-emerging and traditional fieldwork settings [6, 9]. However, students have the impression that role-emerging fieldwork placements are not adequate preparation for clinical practice [8, 9, 18, 19]. Further research on students’ experiences in various role-emerging settings is critical to creating beneficial learning environments that translate to student readiness and self-confidence as occupational therapists [7–9]. Our qualitative study explored occupational therapy students’ perceptions of their fieldwork site, identifying three main themes described in the following paragraphs.

*Integrating Independence and Support is a key* theme that reflects the duality between the independence afforded and the need for support within role-emerging fieldwork. The independence afforded by the distance supervision fieldwork placement was perceived as beneficial. This theme also reinforces previous studies that found that students reported increased self-confidence following role-emerging fieldwork placements [5, 6, 9, 16, 17]. Our participants attributed their confidence building to the support they received from their fieldwork educators through weekly feedback meetings and having a peer partner with whom to debrief. This allowed them to feel supported despite the distant supervision model. This seems unique, as previous studies primarily identified role-emerging fieldwork students feeling a lack of supervisor support which affected their overall feelings of success [8, 16, 18, 19, 28]. The elements of the supervision that were deemed beneficial by the participants included feeling trusted with the responsibilities given, their supervisor’s availability to answer questions outside of the eight hours a week of preceptorship, the effectiveness of the weekly supervision meeting, and the supportive nature of the fieldwork educators. These elements are found in other role-emerging fieldwork literature especially when a collaborative model is used [19, 29, 30], thus these findings are unlikely to fully explain the positive perspective of these students regarding their experience in their host role-emerging fieldwork site.

Our study’s findings affirm recent studies that found that having a peer partner, or collaborative learning, was perceived as essential in role-emerging fieldwork where a fieldwork educator is not available full-time [17–19, 28, 31, 32]. The collaborative learning model has been cited as a successful tool to increase students’ problem-solving and communication skills and develop their ability to be a leader and work in a team [4, 33]. Participants in this study noted these benefits and recognized the transferability of these skills to their future career. Studies have frequently identified the importance of a peer partner in role-emerging fieldwork settings [4, 9, 16, 19, 29, 30, 32, 33], but only a few studies have noted the increase in students’ overall confidence as a result of collaborating with a peer partner [17, 28]. In addition, participants reported that the low-pressure nature of this placement was a further catalyst for building self-confidence and autonomy. Research to date has echoed this point, showing that students in role-emerging placements found that without having an established role of occupational therapy at their site, their professional reasoning skills could grow without constraints [8, 9, 16].

The *Becoming an Occupational Therapist* theme emerged as participants spoke of the skills they learned in this setting and the value of these skills for their future occupational therapy practice. This is important since the literature to date has shown mixed student perceptions on the transferability of skills learned in role-emerging fieldwork to more traditional occupational therapy settings [8, 9]. The occupational therapy fieldwork students in this study did not express this ambivalence, clearly expressing that the skills they learned (e.g., communication, interviewing, professionalism, and time management) could be applied in their future practice. Among other skills, they most frequently noted that developing strong therapeutic relationships with clients was a major takeaway from this placement, a finding that aligns with past research findings [9, 16, 32, 33]. Given that in this role-emerging setting, participants learned to coach, a skillset that, in part, builds one therapeutic use of self, it is surmised that those were some of the skills participants found transferable [34]. Future studies should explore whether this transferability of the coaching skills was actuated.

According to the participants, a major factor that promoted the building of strong therapeutic relationships was being afforded the time to grow and the freedom from the productivity and payor requirements associated with traditional occupational therapy settings. At this fieldwork site, billing was not a factor, and prior research suggests that students appreciate the general freedom to treat using their autonomy and clinical and professional reasoning without the need to meet billing standards or emulate their fieldwork educator [5, 32]. The participants in this study also reported that they developed time management skills in this setting. Time management is a skill that students have identified as facile and challenging in role-emerging placements [9, 32]. Additionally, participants noted that they gained valuable skills in program development and management of occupational therapy services that will be essential to their future practice. Previous studies have cited program development as a key aspect of role-emerging settings [4, 6, 7]. However, Lau and Ravenek [16], found that students reported having difficulties establishing the occupational therapy role and finding their place within an organization during role-emerging fieldwork placement.

Within this theme, the need and opportunity to *learn to advocate* emerged from the data, with participants commenting on the need to advocate on behalf of their clients but also to help other services on campus understand the distinct value of occupational therapy. This development of advocacy skills may be a benefit of this setting and of role-emerging fieldwork settings at large, given that this finding is consistent in the literature [17]. In this fieldwork placement, advocacy came in many forms, but projects were frequently mentioned by participants as opportunities to advocate. The literature has yet to link projects with the opportunity to advocate, such as what was described by participants in this study. Advocacy is closely related to the *Filling a Gap* theme, which highlights the opportunity for occupational therapy to address an untapped need unfilled by other services. During their time in the GOALS^2^ Program, the participants were able to understand the impact of occupational therapy on client progress and success, the wide scope of occupational therapy service provision needed, and the way occupational therapy fits into a university setting.

### Limitations

The sample size (*n* = 14) was appropriate for a qualitative study, and data saturation was reached. Transferability of the study results should be done cautiously for a few reasons. First, all participants had completed a Level I role-emerging placement and may have thus been better prepared for this fieldwork experience. They also all completed their Level II fieldwork within the same role-emerging site and with the same fieldwork educators, limiting our ability to differentiate between the impact of the educational practices used within this novel fieldwork placement vs the experience and skills of the fieldwork educators themselves. It should be noted that both fieldwork educators had limited experience in that role at the beginning of the study. Finally, half of the participants in this study were second career students. It is possible that their previous professional experience influenced their perception of this fieldwork placement. Efforts were also made to minimize the risk of researcher bias (i.e., reflexive journaling, an audit trail, multiple coders, independent thematic analysis, member checking, and peer debriefing with the lead researcher); however, the results may still be inadvertently biased.

### Future directions

A valid and reliable questionnaire of fieldwork students’ perceptions of role-emerging settings could provide additional perspectives from a larger number of students. Additionally, a study comparing the knowledge and skills of practicing clinicians who completed a fieldwork placement in a role-emerging setting in comparison to those who did not would benefit the field.

## Conclusions

Role-emerging practice settings bring the profession back to its roots of occupation as a means of intervention. They meet crucial societal needs and are here to stay, and as a result, occupational therapy students need to develop the unique skills such settings require. The results of this study indicate that occupational therapy students can overwhelmingly perceive role-emerging fieldwork settings as valuable. They saw opportunities to learn skills and know when to seek support, to collaborate intraprofessionally and interprofessionally, to develop programs, and to advocate for the profession, all of which can be used in traditional practice. Some of the features of this placement that are thought to have contributed to the students’ positive experience include the level of support provided, time available for learning including space to make mistakes, and freedom from productivity and payor requirements. Building on the current literature, the findings of this study can be used within future role-emerging placements to enhance students' experience.

## Data Availability

The datasets used and/or analyzed during the current study are available from the corresponding author upon reasonable request.

## Abbreviation

United States: US
